# Endocannabinoid Gene × Gene Interaction Association to Alcohol Use Disorder in Two Adolescent Cohorts

**DOI:** 10.3389/fpsyt.2021.645746

**Published:** 2021-04-20

**Authors:** Laurent Elkrief, Sean Spinney, Daniel E. Vosberg, Tobias Banaschewski, Arun L. W. Bokde, Erin Burke Quinlan, Sylvane Desrivières, Herta Flor, Hugh Garavan, Penny Gowland, Andreas Heinz, Rüdiger Brühl, Jean-Luc Martinot, Marie-Laure Paillère Martinot, Frauke Nees, Dimitri Papadopoulos Orfanos, Luise Poustka, Sarah Hohmann, Sabina Millenet, Juliane H. Fröhner, Michael N. Smolka, Henrik Walter, Robert Whelan, Gunter Schumann, Zdenka Pausova, Tomáš Paus, Guillaume Huguet, Patricia Conrod, the IMAGEN consortium

**Affiliations:** ^1^Department of Medicine, Université de Montréal, Montreal, QC, Canada; ^2^Centre Hospitalier Universitaire Sainte-Justine Research Center, Montreal, QC, Canada; ^3^Department of Pediatrics, Université de Montréal, Montreal, QC, Canada; ^4^Bloorview Research Institute, Holland Bloorview Kids Rehabilitation Hospital, Toronto, ON, Canada; ^5^Department of Child and Adolescent Psychiatry and Psychotherapy, Medical Faculty Mannheim, Central Institute of Mental Health, Heidelberg University, Heidelberg, Germany; ^6^Discipline of Psychiatry, School of Medicine and Trinity College Institute of Neuroscience, Trinity College Dublin, Dublin, Ireland; ^7^Centre for Population Neuroscience and Precision Medicine (PONS), SGDP Centre, Institute of Psychiatry, Psychology & Neuroscience, King's College London, London, United Kingdom; ^8^Department of Cognitive and Clinical Neuroscience, Central Institute of Mental Health, Medical Faculty Mannheim, Heidelberg University, Heidelberg, Germany; ^9^Department of Psychology, School of Social Sciences, University of Mannheim, Mannheim, Germany; ^10^Departments of Psychiatry and Psychology, University of Vermont, Burlington, VT, United States; ^11^Sir Peter Mansfield Imaging Centre School of Physics and Astronomy, University of Nottingham, Nottingham, United Kingdom; ^12^Charité—Universitätsmedizin Berlin, Corporate Member of Freie Universität Berlin, Humboldt-Universität zu Berlin, Berlin, Germany; ^13^Department of Psychiatry and Psychotherapy, Berlin Institute of Health, Campus Charité Mitte, Berlin, Germany; ^14^Physikalisch-Technische Bundesanstalt, Berlin, Germany; ^15^Institut National de la Santé et de la Recherche Médicale, INSERM U1299 “Trajectoires développementales en psychiatrie,” Université Paris-Saclay, Ecole Normale supérieure Paris-Saclay, CNRS, Centre Borelli, Gif-sur-Yvette, France; ^16^Institut National de la Santé et de la Recherche Médicale, INSERM U A10 “Trajectoires développementales en psychiatrie,” Université Paris-Saclay, Ecole Normale supérieure Paris-Saclay, CNRS, Centre Borelli and AP-HP. Sorbonne Université, Department of Child and Adolescent Psychiatry, Pitié-Salpêtrière Hospital, Paris, France; ^17^Neurospin, Commissariat à l'Energie Atomique, CEA-Saclay Center, Paris, France; ^18^Department of Child and Adolescent Psychiatry and Psychotherapy, University Medical Centre Göttingen, Göttingen, Germany; ^19^Department of Psychiatry and Neuroimaging Center, Technische Universität Dresden, Dresden, Germany; ^20^School of Psychology and Global Brain Health Institute, Trinity College Dublin, Dublin, Ireland; ^21^PONS Research Group, Department of Psychiatry and Psychotherapy, Campus Charite Mitte, Humboldt University, Berlin, Germany; ^22^Leibniz Institute for Neurobiology, Magdeburg, Germany; ^23^Departments of Physiology and Nutritional Science, Hospital for Sick Children, Toronto, ON, Canada; ^24^Departments of Psychology and Psychiatry, University of Toronto, Toronto, ON, Canada; ^25^Department of Psychiatry, Université de Montréal, Montréal, QC, Canada

**Keywords:** alcohol use disorder, cannabinoid receptor 1, CNR1, DAGL, endocannabinoid system, MGLL

## Abstract

Genetic markers of the endocannabinoid system have been linked to a variety of addiction-related behaviors that extend beyond cannabis use. In the current study we investigate the relationship between endocannabinoid (eCB) genetic markers and alcohol use disorder (AUD) in European adolescents (14–18 years old) followed in the IMAGEN study (*n* = 2,051) and explore replication in a cohort of North American adolescents from Canadian Saguenay Youth Study (SYS) (*n* = 772). Case-control status is represented by a score of more than 7 on the Alcohol Use Disorder Identification Test (AUDIT). First a set-based test method was used to examine if a relationship between the eCB system and AUDIT case/control status exists at the gene level. Using only SNPs that are both independent and significantly associated to case-control status, we perform Fisher's exact test to determine SNP level odds ratios in relation to case-control status and then perform logistic regressions as *post-hoc* analysis, while considering various covariates. Generalized multifactor dimensionality reduction (GMDR) was used to analyze the most robust SNP×SNP interaction of the five eCB genes with positive AUDIT screen. While no gene-sets were significantly associated to AUDIT scores after correction for multiple tests, in the case/control analysis, 7 SNPs were significantly associated with AUDIT scores of > 7 (*p* < 0.05; OR<1). Two SNPs remain significant after correction by false discovery rate (FDR): rs9343525 in *CNR1* (p_corrected_ =0.042, OR = 0.73) and rs507961 in *MGLL* (p_corrected_ = 0.043, OR = 0.78). Logistic regression showed that both rs9353525 (*CNR1*) and rs507961 (*MGLL*) remained significantly associated with positive AUDIT screens (*p* < 0.01; OR < 1) after correction for multiple covariables and interaction of covariable × SNP. This result was not replicated in the SYS cohort. The GMDR model revealed a significant three-SNP interaction (*p* = 0.006) involving rs484061 (*MGLL*), rs4963307 (*DAGLA*), and rs7766029 (*CNR1*) predicted case-control status, after correcting for multiple covariables in the IMAGEN sample. A binomial logistic regression of the combination of these three SNPs by phenotype in the SYS cohort showed a result in the same direction as seen in the IMAGEN cohort (BETA = 0.501, *p* = 0.06). While preliminary, the present study suggests that the eCB system may play a role in the development of AUD in adolescents.

## Introduction

Substance use disorders are a growing concern across the world, with an estimated 31 million users worldwide suffering from drug use disorders. After alcohol and tobacco, cannabis ranks as the most used drug worldwide (1). Moreover, those who use cannabis are more than five times more likely to have an alcohol use disorder (AUD) (2). Considering that the endocannabinoid (eCB) system is responsible for the physiological consequence and subjective “high” of cannabis, much attention has been paid to the eCB role in the development of various substance use disorders. Cannabinoid receptors and related enzymes are expressed in many of the reward centers of the brain: nucleus accumbens (NAc), ventral tegmental area (VTA), amygdala, and basal nucleus of the stria terminalis (BNST) (3, 4). These eCB levels are affected by ethanol (5), and the eCB system plays a role in the development of AUD and other substance use disorders in humans (4). Basavarajappa and colleagues (6) demonstrated that acute ethanol use has been associated with an increase in eCB signaling, while others have reported that alcohol use decreases eCB signaling (7, 8). Moreover, as is the case with other drugs of abuse, eCBs mediate the reward signals associated with alcohol use (9). Overall, the underlying evidence shows that the eCB system is modulated by ethanol use, and this same system may play an independent role in AUD (10).

The first eCB receptor isolated, of which tetrahydrocannabinol (THC) is also a ligand, is the cannabinoid receptor one (CB1) (11, 12). Binding to this receptor and a second cannabinoid receptor (CB2) are the two main eCB agonists, anandamide (AEA) and 2-arachidonoylglycerol (2-AG). These agonists—which are not stored in vesicles—are produced through an enzymatic cascade in a Ca^22++^ dependent manner, and then are rapidly degraded by specific enzymes, fatty acid amide hydrolase (FAAH) and monoacylglycerol lipase (MAGL). N-acylphosphatidylethanolamine-specific phospholipase D (NAPE-PLD) plays a crucial role in the synthesis of AEA, which then binds to CB1. 2-AG is synthesized by diaglycerol lipase (DAGL).

It has been shown that polymorphisms in the *CNR1* gene, the gene coding for the CB1 receptor protein, are associated with a range of diseases, psychiatric disorders, and substance use (13–15). Many studies have assessed the various aspects of the eCB genes and their relationship with substance use disorders and risk-taking behavior. The single nucleotide polymorphism (SNP) rs1049353 in the *CNR1* gene has been associated with severe alcoholism (minor A allele) (16), heroin addiction (major G allele) (17), and impulsivity (18). Furthermore, haplotype blocks within the *CNR1* gene have been associated with addiction and addictive behavior (19, 20). Polymorphisms in the *FAAH* gene have also been associated with problem drug use and addiction (21, 22). In contrast, there have been relatively few studies examining the *MGLL* gene, the gene coding for the MAGL enzyme, and the *DAGL* in association with drug dependence (23–25). Among these, only one study has found a positive association between SNPs of the *MGLL* gene and drug dependence (25), while no studies have reported a significant association between *DAGL* and any form of drug abuse. Moreover, many of the original findings reporting an association between SNPs located in genes of the eCB and various drug abuse behaviors have not been replicated (4, 26), suggesting the possibility of false positive results in these candidate gene approaches. Nevertheless, while there are conflicting results among studies, the candidate gene literature suggests that genes related eCB proteins may play a role in the development of substance use problems.

While candidate gene findings in psychiatric genetics have been widely criticized for replication failure, particularly with respect to GWAS and meta-analysis (27), candidate gene approaches in addiction research have identified genetic markers that have been confirmed in GWAS and meta-analysis (28). This is perhaps related to particularly heritable nature of addictive behaviors compared to other psychiatric conditions, or to the fact that candidate gene approaches can be directly informed by pharmacogenetic studies on how drugs of abuse interact with the brain's neurochemistry. Others have argued (29) that the failure to replicate candidate gene findings through GWAS and meta-analysis does not necessarily suggest the findings are false. The candidate gene findings may represent particular endophenotypes of sub-populations, which may account for a portion, albeit small, of genetic influence on the phenotype in question. Thus, other groups have utilized novel methodologies, such as gene-set approaches, to analyze hypothesis-based questions in psychiatric genetics and addiction medicine. Recently, one group, utilizing said gene-set approaches, found that *MGLL* and the SNP rs604300 interact with childhood sexual abuse to predict cannabis dependence symptoms (25). Considering our relatively limited understanding of the roles of the various endocannabinoid genes in the pathogenesis of addictive behaviors, and the lack of robust findings at the individual SNP, or GWAS levels, gene-set, and system-based approaches remain of interest (25, 30). Thus, the current study employs a gene-set based approach in an attempt to shed light on the role of the eCB system in the pathogenesis of addictive behaviors.

Given the effect of alcohol on the eCB system (5) and the purported relationship between eCB SNPs and the risk for substance use disorder, we assessed the association between eCB genetics and alcohol abuse behaviors in the IMAGEN cohort (31). The IMAGEN cohort is a European cohort of 2,087 adolescents recruited in France, UK, Ireland, and Germany. Endocannabinoid genetic influence was studied through a candidate gene approach. Multiple SNPs in eCB genes that have been previously examined (*CNR1, FAAH, MAGL, DAGLA*) as well as genes that have not yet been investigated (*NAPEPLD*) were analyzed in the context of alcohol use disorder (AUD). To understand this relationship, a three-tiered approach was used. First, a set-based test (32) is utilized to study, at the gene level, the link between the eCB system and alcohol abuse behavior. Through this approach we also identify SNPs that are significantly and independently associated to positive AUD screening, and these SNPs are selected for further study using a case/control analysis and subsequent logistic regression. Finally, while some studies have investigated the interaction between two eCB genes and addictive behavior (33, 34), none have examined the eCB system as a whole. Considering the complex interplay between the multiple eCB ligands (AEA and 2-AG among others) and various receptors (CB1, CB2, etc.) in their relationship to addictions (4), we hypothesize that a single genetic marker association study could not account adequately for the multifaceted role the eCB system plays in risk for AUD. A new wave of candidate gene studies have explored more complex gene-gene interactions, using various methods of multifactor dimensionality reductions analyses to yield promising results such as predicting outcomes in breast cancer treatment (35), in determining genetic biomarkers to predict antidepressant response (36), and further understanding the genetic influences of nicotine addiction (37). Here, we utilized Generalized Multifactor Dimensionality Reduction (GMDR) to understand the effects that the multiple eCB genes may have on each other and their combined influence on alcoholic behavior in adolescence. To replicate the results, genetic and alcohol use data were used from the Saguenay Youth Study (SYS), a two-generational study comprised of 1,029 French-Canadian adolescents and their parents.

## Materials and Methods

### Participants

The IMAGEN study is a longitudinal imaging genetics study of 2,087 healthy adolescents, mostly of European descent. Detailed descriptions of this study, genotyping procedures, and data collection have previously been published (31). The IMAGEN cohort has been repeatedly assessed on substance use outcomes at 14, 16, and 18 years of age. The multicentric IMAGEN project had obtained ethical approval by the local ethics committees (at their respective sites) and written informed consent from all participants and their legal guardians. The parents and adolescents provided written informed consent and assent, respectively. All datasets were de-identified by using codes for individuals. See Schumann et al. (31) for a more detailed description of the IMAGEN cohort.

The current study used data for all 2,087 individuals who completed the IMAGEN assessment battery at 14, 16, and 18 years of age and who contributed their genetic data at 14 years of age. Of those followed at 16 or 18 years of age, three individuals had unassigned sex according to sex determination analysis in PLINK1.9 (38) and were thus excluded from the genetic analyses. Moreover, 11 individuals did not answer the Alcohol Use Disorder Identification Test (AUDIT) at any time point and were thus removed from the genetic analyses. Eleven pairs of siblings were a part of the IMAGEN database, and thus one sibling from each pair was removed from the study, according to the methods published (39). European ancestry was determined using Admixture (40) using HapMap III (41) as a reference population. Eleven individuals with non-European ancestry were removed prior to analysis. Thus, in this study there was a total of 1,043 female and 1,008 males. A summary of the individuals can be seen in Table 1 and Supplementary Table 1.

**Table 1:** Description of subjects in IMAGEN and SYS.

**Cohort**		**IMAGEN**	**SYS**
N (female %)		2051 (50.8%)	772 (52.07%)
N family		2051	401
Age (SD)		14 to 18 years^c^	15 years (1.85)
AUDIT^a^	Control	1476	-
	Case	575	-
GRIP^b^	Control	-	724
	Case	-	48

a *IMAGEN subjects are classified by status with AUDIT score, case is > or = to 8 and control < 8*;

b *SYS subjects are classified by status with GRIP score, case > or = to 2 and control < 2*.

c *IMAGEN cohort is a longitudinal cohort, so it's not possible to calculate the standard deviation (SD)*.

### Phenotype Evaluated

#### Alcohol Misuse

AUDIT is a self-report questionnaire developed by the World Health Organization and validated (42) to screen for heavy drinking and current alcohol dependence. Individuals were considered to screen positive for risk for AUD and were included in the case group if they scored 8 or more on the AUDIT (case-control status). While other studies focusing on adolescent alcohol abuse used a less stringent cut-off (43–45), the more stringent cut-off of 8 was chosen as this is the cut-off with the strongest sensitivity and a favorable specificity across all studies (46). Four AUD scores were derived: “Any AUD” representing having screened positive for AUD at any timepoint from 14–18 years of age, and then individual dichotomized scores for each of the time points, 14, 16, and 18 years. For details about choice of cut-off, see Supplementary Methods.

#### Covariates

Covariables include sex, the first six genetic principal components, parental alcohol abuse, and parental education. Parental education was taken from the parent-report questionnaire using the educational categories specified in the European School Survey Project on Alcohol and Other Drugs (ESPAD+) questionnaire. Risk for AUD in parents was measured using the AUDIT obtained at the first two time points in IMAGEN. If ESPAD+ and AUDIT information were missing at the 18-year-old time point, the most complete and recent information was used at this time point. If a parent had signaled a positive AUDIT at any time, they were flagged as such. Moreover, if parental information was missing, individuals were not included in the logistic regression.

### Pipeline of SNP Selection

The genotyping was run using the Illumina Quad 610 chip and 660Wq at the “Centre National de Genotypage” (Paris, France). Only autosomal SNPs were kept for this study. SNPs with a minor allele frequency (MAF) of <5%, a missing SNP rate of 10%, or SNPs that did not respect Hardy Weinberg Equilibrium (HWE) (<1 × 10^−6^) were also removed from this study. All available SNPs in the genes of interest (*CNR1, NAPE, FAAH, MGLL, DAGLA*) within ±10 kb (to include promoter and flanker regions) were then selected. Gene length and location were obtained using the UCSC Genome Browser. The SNP coordinates were updated from hg18 to hg19 using Illumina information and the liftover tool from the genome browser (http://genome.ucsc.edu/cgi-bin/hgLiftOver). Nevertheless, SNP information was scarce on the *CNR2*, and as such, the gene was not included in this study. A summary of the locations and details of each SNP (gene, chromosome, base pair, function, etc.) can be seen in Supplementary Table 2.

### Statistical Analysis

Sixty-nine SNPs appearing across five cannabinoid-related genes were analyzed for their relation to problematic alcohol consumption. As a primary analysis, we first conduct three set-based tests using parameters of varying stringencies, to study the relationship between 5 endocannabinoid gene-sets (*CNR1, NAPEPLE, FAAH, MGLL, DAGLA*). The parameters that were adjusted between the tests were *p*-value for significant variants between tests, *r*^2^ of variant pairs, and maximum set size. Data in all three set-based tests underwent 10,000 label-swapped permutation as well, using the—perm function in PLINK1.9. The first test was the default test in PLINK1.9, with a *p*-value of 0.05, *r*^2^ of 0.5, and a set-max of 5; the second test had a *p*-value of 0.05, *r*^2^ of 0.3, and set-max of 3; while test 3 had a *p* of 0.01, *r*^2^ of 0.1, and set-max of 2. Tests 2 and 3 were more stringent and were run to challenge the data, to ensure robustness of our results. Statistical significance for set-based test was determined using a Bonferroni corrected empirical *p*-values of *p* < 0.01 (0.05/5 genes). Burden and optimized sequence kernel association tests (SKAT, R package) (47) were used to analyze the joint effects of SNPs (in gene sets). These analyses were performed on three groups of variants: (a) Set 1, (b) Set 2, (c) Set 3 defined with gene set PLINK analyses. We resampled 10,000 times to compute empirical *p*-values (*p*-values were adjusted controlling for family-wise error rate) for the analyses (with “bootstrap” option).

Next, to determine SNP level odds ratios (OR) case-control analysis was run on the SNPs that were nominally significant and independent after set-based analysis, using Fisher's exact test. In the case-control analysis, false discovery rate (FDR) was used to correct for multiple tests. To test the robustness of these findings after controlling for various relevant covariates, a logistic regression was performed that included only the SNPs that remained significant after correction for multiple tests, sex, the first six ancestry components, parental AUDIT flag, and parental education were included in the logistic model. In *post-hoc* analysis, for SNPs that significantly predicted case-control status, after controlling for covariates, we control for potential confounding of interaction (48) and include the interaction of the covariate of no interest by SNP (see Supplementary Methods for descriptions of the covariables and Supplementary Figure 1 for results of principal component analysis). The set-based test, Fisher exact test, and logistic analyses were all carried out using PLINK program (38).

### Generalized Multifactor Dimensionality Reduction

In order to test the replicability of these findings across a different analytic strategy, GMDR was employed to analyze the SNP x SNP interaction with phenotype. GMDR (v1.0) is a free open source tool for identification of interactions, developed by Guo-Bo Chen (49). This program was used to screen for the best interaction combinations among the 69 SNPs and the phenotype of interest. Permutation with 10,000 shuffles providing empirical *p*-values to measure the significance of an identified model was used. For these analyses, logistic regression with the same covariables as described above were performed. For more information on the GMDR method see Lou et al. (49).

### SYS Replication Cohort

Genetic and alcohol-use data from the Saguenay Youth Study (SYS) were used to replicate the findings. The SYS is a two-generational study comprised of 1,029 adolescents and 962 parents (50). For descriptive characteristics of the participants included in the replication see Supplementary Table 3 and Table 1. All individuals were genotyped using whole blood samples from which DNA was extracted. The genotyping was performed at “Centre Nationale de Génotypage” for 610Kq (No. arrays = 599) and at the Genome Analysis Centre of Helmholtz Zentrum München (Munich, Germany) for HOE-V12 (No.arrays = 1,395). Genetic information was imputed following previously published methods (50) and after that, the 69 SNPs studied were extracted. Detailed descriptions of the cohort, genotyping, and data collection have previously been published (50, 51).

Participants were recruited over a 10-year period. Once recruited, adolescents provided genetic material and underwent a detailed assessment in several domains. Alcohol-use data for the SYS cohort were obtained *via* a self-report questionnaire developed specifically for the SYS to assess mental health and substance use based on validated protocols (52). The items from this questionnaire that were deemed to overlap sufficiently with AUDIT questions are listed in Supplementary Table 4. Of 1,029 adolescents in the SYS cohort, 772 adolescents aged 14 years and older had completed both the SUD assessment and provided genetic information, and were therefore included in this study.

In the replication of the case-control study, we studied the 7 SNPs found in the set-based test. Description of SNPs can be found in Supplementary Table 5. Two statistical models were used to study the replication group. To study the native continuous phenotype, a model based on the quasi-poisson distribution was used. The participants were also separated into four different drinking groups, based on scoring distribution. A binomial logistic model was then used separating the participants into controls (groups 0–1; low alcohol use) and cases (groups 2–4; high alcohol use). Both models considered sex, age as covariables and family ID as random effect. Statistical analyses were performed using R, with the glmmTMB library, version 3.5.3 (https://www.R-project.org/).

## Results

### Set-Based Tests: Identifying Candidate SNP

The three set-based tests were run, with varying results (Supplementary Table 6). In the first set-based test, nine SNPS returned with nominal *p*-values of <0.05, of which seven also passed linkage disequilibrium (LD) criterion. Through the first set-test criterion, only the CNR1 gene-set had a significant empirical *p*-value (*p* = 0.022), but this was not significant after correction for multiple tests. Within this set, only rs9353525 was significantly and independently related to dichotomized AUDIT scores. In the second set-based test, the same nine SNPS returned with nominal *p*-values of <0.05, of which five SNPs passed the LD criterion. Nonetheless, no gene sets were significantly associated to case control status (*p* > 0.05). Finally, four SNPs returned with a *p*-value <0.01 in the third test, with two SNPs passing LD criterion. No genes remained significant after correction for multiple testing (p_FDR_ > 0.05). As mentioned above, the seven SNPs that had marginal *p*-values of <0.01 in the first set-based test, and that passed LD criterion (*r*^2^ < 0.5), were extracted, and only these were analyzed in the case-control analysis and logistic regression analysis. SKAT demonstrated similar results for the CNR1 gene (Supplementary Table 7).

### Case-Control Analysis and Sensitivity Analysis

In the case-control analysis of the IMAGEN cohort, which considered cases as individuals who scored eight or more on AUDIT at any time point (ALL), all 7 SNPs analyzed were significant (*p* < 0.05) (Table 2). All of the minor alleles were protective against having a case control status (OR < 1). Two SNPs remained significant after correction by FDR: rs9343525 in *CNR1* (p_FDR_ = 0.043, OR = 0.73) and rs507961 in *MGLL* (p_FDR_ = 0.043, OR = 0.78). A multivariate logistic regression analysis was done for the two SNPs that were significant after FDR correction in the Fisher test (Table 3). As a first *post-hoc* analysis logistic models were done for significant SNPs, at each time point (14, 16, and 18), as well as for any positive screen (ALL) for case-control status. After controlling for the effects of the first six principal components, sex, parental AUDIT scores (at any time), and parental education, both rs9353525 and rs507961 were still significantly associated with positive AUDIT screen in the ALL analysis (Table 3) (*p* < 0.01), with both SNPs minor allele acting as protective factors (OR < 1). In our *post-hoc* analysis, we find a significant interaction between rs9353525 and PC1 and PC6, as well as a significant interaction of rs507961 and PC3, suggesting that the genetic background, captured by the principal components, may modify the genetic effects of the SNPs on AUDIT scores. For complete results of logistic regression see Supplementary Table 9, and see Supplementary Table 10 for results of *post-hoc* interaction analyses. Finally, we conducted *post hoc* analyses to study the association between AUDIT scores and SNPs of interest at each IMAGEN time point (14, 16, and 18 alone). After correction for multiple testing, none of the *post-hoc* analysis demonstrated significant results (see **Supplementary Results** for detailed results).

**Table 2:** Table of results for case/control analysis ALL.

**SNP**	**A1**	**A2**	**Freq AC**	**Freq AU**	**OR**	**Pvalue**	**FDR Pvalue**
rs782446	C	A	0.22	0.26	0.83	0.024	0.081
rs484061	G	A	0.46	0.51	0.83	0.0091	0.055
rs604300	A	G	0.09	0.11	0.77	0.033	0.085
rs507961	T	C	0.20	0.24	0.78	0.0047	0.043
rs9353525	A	G	0.10	0.14	0.73	0.004	0.043
rs4729873	G	A	0.33	0.38	0.85	0.027	0.081
rs10488693	T	C	0.06	0.08	0.73	0.026	0.081

*A1, minor allele; A2, major allele; Freq AC, frequency of minor allele in cases; Freq AU, frequency of minor allele in controls; OR, odds ratio. FDR Pvalue, p-value after false discovery rate correction*.

**Table 3:** Table of results for logistic model with AUDIT and rs9353525 and rs507961.

**Phenotype**	**SNP**	**A1**	**NMISS**	**BETA**	**OR**	**STAT**	***P***
AUDIT ALL	rs507961	T	2030	−0.27	0.76	−3.06	**0.002**
	rs9353525	A	2026	−0.30	0.74	−2.61	**0.009**
AUDIT for 14	rs507961	T	2024	−0.24	0.78	−1.10	0.27
	rs9353525	A	2020	−0.22	0.81	−0.78	0.44
AUDIT for 16	rs507961	T	1535	−0.19	0.83	−1.49	0.14
	rs9353525	A	1532	−0.46	0.63	−2.49	**0.01**
AUDIT for 18	rs507961	T	1243	−0.30	0.74	−2.59	**0.01**
	rs9353525	A	1240	−0.32	0.73	−2.05	**0.04**

*A1, minor allele. NMISS, number of non-missing individuals. OR, odds ratio. Stat, coefficient t-statistic. p < 0.05*.

In the replication cohort, rs484061 was significantly associated with problematic alcohol use (*p* = 7.47^*^10^−6^) in the binomial model. None of the other SNPs in the replication analysis had a significant result, after correction for multiple tests (Supplementary Table 8).

### GMDR: SNP × SNP Interactions

**Figure 1:**
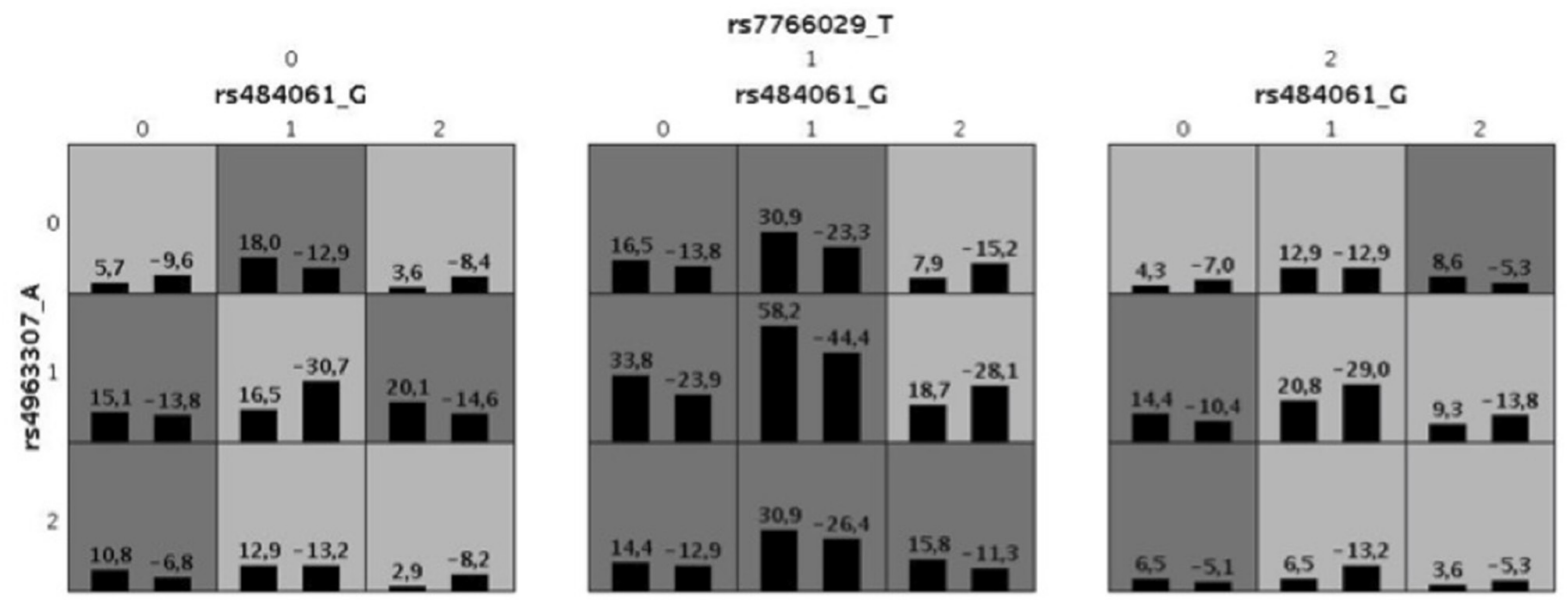
Illustration for the best combination defined by GMDR for 69 SNPs for AUDIT. The allele code is defined by minor allele numbers of rs484061 (allele G), rs4963307(allele A) and rs7766029 (allele T). The numbers above the histogram bar, indicate the sum of “positive” (above the averaged score = 0) and “negative” (below the averaged score = 0) scores by the combination of genotypes. Also, the dark gray indicates a high-risk combination of the genotypes with alcoholism and light gray for low risk. It defined by sum of positive and negative score, when it's < 0 for low-risk and > 0 for high-risk.

A GMDR model was used to screen for the most robust interaction of combinations for the 69 SNPs in the candidate genes and case control status. For the one and two-SNP models, no significance was found p>0.05. However, we found a significant three-SNP model (*p* = 0.006) involving rs484061 (*MGLL*, intron), rs4963307 (*DAGLA*, intron), and rs7766029 (*CNR1*, downstream-gene) with AUDIT positive screens. An interaction between rs484061, rs4963307, and rs7766029 was significantly associated with case-control status, with a combination of G/A;G/A;C/C or G/G;G/G;C/C conferring protection against problem drinking in the cohort (*p* = 0.004 and *p* = 0.02, respectively; Supplementary Table 11). The cross-validation consistency of this three-locus model was 19/20. The testing accuracy of the three SNP model (54%) was greater than the testing accuracy of either the one SNP (49%) or two SNP models (50%) (Table 4 and Figure 1). This result was verified by re-analyzing the model using 10 different random seeds, and this model remained significant for each seed. An analysis of the same three SNP combination in the SYS cohort, binomial logistic model, showed a result in the same direction as seen in the IMAGEN cohort (BETA = 0.50, *p* = 0.06), and the distribution of at risk and protective combinations of SNP with phenotype is comparable to that of the IMAGEN population (Supplementary Tables 11, 12).

**Table 4:** Table of results for the best combinations defined by GMDR for 69 SNPs for AUDIT.

**Model**	**Training accuracy**	**Testing accuracy**	**Sign test (*p*)**	**CV consistency**
[rs806368]	0.53	0.49	8 (0.94)	15/20
[rs806368, rs10488693]	0.55	0.50	12 (0.17)	15/20
[rs484061, rs4963307, rs7766029]	0.58	0.541	16 (0.006)	19/20

*Model, SNPs included in the model; Sign test, sign test result with p-value in parentheses; CV consistency, cross validation consistency*.

## Discussion

Although no gene-sets were significantly predictive of binary AUDIT scores, after correction for multiple tests, our case/control analysis suggest that two SNPs, rs507961 (*MGLL*) and rs9343525 (*CNR1*), are associated with problem drinking and remained significantly associated after correction for multiple tests. The SNPs remained significantly associated to case-control status in logistic regression, while considering multiple covariables, and the interaction of these covariables and the SNPs in question. The results of our logistic regression were not replicated in the replication cohort. To our knowledge, one study (25) had investigated rs507961 in *MGLL* in relation to substance use disorders; however, the association did not remain significant after correction for multiple tests. While rs507961 is intronic in *MGLL*, this SNP plays a role in histone regulation of this gene in the brain (Supplementary Table 13). The robustness of our result confers evidence that carrying the minor T allele may in fact confer protection against problem drinking. Moreover, no study has investigated the relationship between rs484061, another *MGLL* SNP, and substance use disorders. The recurrence of rs484061 in both the GMDR model and case-control analyses suggests that being a carrier of this SNP protects against risk for AUD. While rs484061 was significantly associated to positive AUDIT screens in the case-control analysis of the IMAGEN cohort (*p* = 0.009, pFDR = 0.055) and replicated in SYS (*p* = 7.47^*^10^−6^), it was significantly associated to lower alcohol use. Our results suggest a role for *MGLL* in AUD but work in larger cohorts is needed to confirm this result.

The second SNP that remained significant after correction for multiple tests in our case-control analysis was rs9353525. It is localized in an intergenic region <10 Kb of the 3′ region of *CNR1*. In an attempt to understand the biological role that this SNP plays in the regulation of *CNR1* expression, we scanned the various available databases for potential roles; however, this SNP is relatively understudied. While this SNP was not associated with higher rates of alcohol use in the SYS cohort, this SNP is in strong linkage disequilibrium with rs806368 (at 78% for allele T with G respectively for rs806368 and rs9353525). The rs806368 has been associated to alcohol dependence in other studies (53). We also investigated rs806368 in our cohort, using the same case-control analysis as for our other SNPs, and the major allele is associated with likelihood of reporting a clinically significant AUDIT score at any timepoint in the IMAGEN cohort (*p* = 0.007 OR = 1.28). Moreover, this result remains significant after controlling for the various covariates described above in the IMAGEN cohort (*p* = 0.007; see Supplementary Tables 14, 15). Taken together, these results suggest that the haplotype block containing both of the major alleles of rs9353525 and rs806368 plays a role in the development of AUD in adolescents.

A GMDR model was used to screen for the gene x gene interaction that would be most associated to problem drinking, across genes showing a signal in previous analyses. We found a significant interaction involving rs484061 (*MGLL*), rs4963307 (*DAGLA*), and rs7766029 (*CNR1*) that predicted clinically significant AUDIT scores after correction for covariates. Each of these three SNPs are associated to loci, which are key regulators of gene expression (Supplementary Tables 13, 16). This observation was supported by the consistency of the result in the GMDR, across IMAGEN and SYS GMDR results (*p* = 0.06) (Supplementary Table 8). The similar distribution pattern of problem drinkers within the SYS cohort suggests that the marginal result in the SYS cohort is probably due to a lack of statistical power. The SYS cohort comprises a relatively young sample (mean age = 15 years old), as compared to the IMAGEN cohort, which includes data from individuals when they are 14, 16, and 18 years of age. As such, many of the participants in the SYS cohort have not had their first contact with alcohol, and therefore might not have developed heavy patterns of drinking. This marginal effect should be investigated using data from this sample as it ages, to explore whether the effect becomes larger and more significant when substance use behaviors are assessed during the typical age when substance use disorders have their onset.

### Endocannabinoid Interactions in the Brain and Emotional Regulation

The GMDR analysis suggests that a certain combination of SNPs along the *CNR1-DAGLA-MGLL* genes protect against or pose a risk for alcoholism, by presumably modulating DAGLA and or MGLL expression and subsequently 2-AG levels. The DAGLA protein (encoded by *DAGLA*) catalyzes the formation of 2-AG, which then acts as an agonist of CB1. Then, 2-AG is promptly degraded by MAGL (encoded by *MGLL*). Also, 2-AG has been shown to play a key role in the regulation of the hypothalamic-pituitary-adrenal (HPA) stress response axis (54), which is altered in alcohol addiction (55). In response to increased corticosterone, 2-AG levels increase in the medial prefrontal cortex and paraventricular nucleus of the hypothalamus, and act as a negative feedback signal to inhibit the HPA axis and terminate the acute stress response (54). While 2-AG levels increase *in situ*ations of chronic stress, it is theorized to play a role in stress habituation (54). Along the same line, 2-AG has also been shown to play a role in the reduction of stress induced-anxiety in a role mediated through the actions of MAGL and DAGLA (54). MAGL antagonists have been shown to have a strong anxiolytic effect in rodents (56, 57). Knockout studies have shown that *DAGLA (-/-)* mice, which have large reductions in brain 2-AG levels, have increased anxiety-like symptoms (58, 59). Moreover, the anxiety-like state seen in animal models of alcohol dependence and withdrawal symptoms are mediated by corticosterone-releasing factor release in the central nucleus of the amygdala (CeA) (60). A recent study in alcohol dependent rodents found that 2-AG levels were decreased in the CeA of these animal models, and that inhibition of MAGL, increasing 2-AG levels, ameliorated abstinence-related anxiety and excessive alcohol intake (61). Mice exposed to chronic mild stress have reduced levels of *DAGLA* expression, and reduced *DAGLA* expression in this same study was significantly associated to increased preference for alcohol (62). The study by Ishiguro and colleagues was also the first to link SNPs in the *DAGLA* gene and alcoholism in humans (62). Our study supports the hypothesis that suggests that the eCB system plays a role in the development and/or maintenance of AUD in adolescents. Previous findings suggest that this vulnerability might be achieved by affecting sensitivity to anxiety-like symptoms and influencing reward sensitivity to alcohol intake and warrants further study.

While the results of this study suggest a relationship between eCB genes and AUD, we must acknowledge that the results of this study are preliminary and modest. First, many researchers have called hypothesis-based candidate gene approaches into question (27, 63, 64). This is due to the fact that, while very large GWAS studies consistently report that individual SNPs exert very small effects on complex phenotypes such as addiction, most published studies in the field report significant results, even with relatively small sample sizes (27). Considering that these small candidate gene studies may be underpowered (65, 66) (including ours), the significant results reported in the past are most likely false positive (27). It is also possible that this might be the case in the current study; however, the use of a replication sample provides a context in which to interpret the findings and make conclusions about generalizability of the findings.

Moreover, we were unable to replicate many of the previously reported findings in relation to substance use and eCB genes. This is because our set-based test eliminated many of the previously reported SNPs as they were non-independent according to our criteria. Moreover, some SNPs that are previously reported, mainly rs2023239 (9, 34) and rs6454674 (53, 67, 68), are not assessed in the assay chips used in the present study or were too infrequent in our cohort for analysis. This was also the case for SNPs within *CNR2* that have been previously evaluated for their relationships with substance use. Considering that our findings were most robust within the analysis considering all timepoints, we cannot be certain what role these SNPs play in the development of AUD (initiation of drinking, susceptibility to binge drinking, proneness toward harmful alcohol use or maintenance of abuse habits, etc.). Our findings suggest a more robust relationship at later time points, potentially related to the power that increased prevalence of AUD at the older age affords in a statistical analysis. However, it will also be important to investigate whether these genetic markers are linked to maintenance of drinking in adults, relative to early initiation behaviors, using larger longitudinal cohorts, when they become available. Finally, there are limitations with the cohort used for this study. Considering that our cohort is population-based sample of adolescents, the number of problem drinkers is relatively low. Moreover, as the cohorts aged, they reduced in size due to participants leaving the study, diminishing the power of the analyses. Finally, while the results of our replication study were in line with the results of the IMAGEN analysis, our main findings were not significant according to classic standards (*p* = 0.05).

Nevertheless, the present suggests an interaction among various candidate genes relevant to the eCB system in predicting AUD, specifically the *CNR1*-*MGLL*-*DAGL* loop and their relationship to 2-AG. Further studies are required to further explore the generalizability of these findings and to understand the psychiatric implications of the results.

## Data Availability Statement

The data analyzed in this study is subject to the following licenses/restrictions: Data for Imagen dataset and SYS can be made available upon request. Requests to access these datasets should be directed to For Imagen dataset, data can be requested at: https://imagen-europe.com/resources/imagen-project-proposal/. For SYS please address: Dr. Zdenka Pausova [zdenka.pausova@sickkids.ca] and Dr. Tomas Paus [tpaus@research.baycrest.org]. Further details about the protocol can be found at [http://www.saguenay-youth-study.org/].

## Ethics Statement

The studies involving human participants were reviewed and approved by Psychiatry, Nursing and Midwifery Research Ethics Subcommittees (PNM RESC), King's College London. Written informed consent to participate in this study was provided by the participants' legal guardian/next of kin.

## Author Contributions

LE conceptualized the analysis, ran the analysis using PLINK1.9, wrote and edited the manuscript. SS helped conceptualize the analysis and ran analyses using the PLINK1.9. DV compiled the SYS cohort data and edited the manuscript. TP helped secure access to the SYS and IMAGEN data, edited the manuscript, and supervised the work. PC and GH supervised the project, helped conceptualize the project, secured access to the data, and edited the manuscript. All authors contributed to the article and approved the submitted version.

## Conflict of Interest

TB served in an advisory or consultancy role for Lundbeck, Medice, Neurim Pharmaceuticals, Oberberg GmbH, Shire. TB received conference support or speaker's fee by Lilly, Medice, Novartis and Shire. TB has been involved in clinical trials conducted by Shire & Viforpharma. TB received royalties from Hogrefe, Kohlhammer, CIP Medien, Oxford University Press. The present work is unrelated to the above grants and relationships. LP served in an advisory or consultancy role for Roche and Viforpharm and received speaker's fee by Shire. LP received royalties from Hogrefe, Kohlhammer and Schattauer. The present work is unrelated to the above grants and relationships. The remaining authors declare that the research was conducted in the absence of any commercial or financial relationships that could be construed as a potential conflict of interest.
