# Supplementary material for: Endocannabinoid Gene × Gene Interaction Association to Alcohol Use Disorder in Two Adolescent Cohorts
Source: Front Psychiatry. 2021 Apr 20;12:645746. doi: 10.3389/fpsyt.2021.645746 (PMC8093566; doi:10.3389/fpsyt.2021.645746)
Supplement: Supplementary file 2 [file Data_Sheet_2.docx]

Endocannabinoid Gene x Gene Interaction Association to Alcohol Use Disorder in two Adolescent Cohorts.

Laurent Elkrief ^1,2^, Sean Spinney ^2, 3^, Daniel E. Vosberg ^4^, Tobias Banaschewski M.D., Ph.D.^5^; Arun L.W. Bokde Ph.D.^6^; Erin Burke Quinlan PhD^7^; Sylvane Desrivières Ph.D.^7^; Herta Flor Ph.D.^8,9^; Hugh Garavan Ph.D.^10^; Penny Gowland Ph.D.^11^; Andreas Heinz M.D., Ph.D.^12^; Rüdiger Brühl Ph.D.^13^; Jean-Luc Martinot M.D., Ph.D.^14^, Marie-Laure Paillère Martinot M.D., Ph.D.^15^; Frauke Nees Ph.D.^5,8^; Dimitri Papadopoulos Orfanos Ph.D.^16^; Luise Poustka M.D.^17^ ; Sarah Hohmann M.D.^5^; Sabina Millenet Dipl.-Psych.^5^; Juliane H. Fröhner MSc^18^; Michael N. Smolka M.D.^18^; Henrik Walter M.D., Ph.D.^12^; Robert Whelan Ph.D.^19^; Gunter Schumann M.D.^7, 20^; Zdenka Pausova^21^, Tomáš Paus ^4, 22^, Guillaume Huguet ^2,3^†, Patricia Conrod ^2,3,23*^†, and the IMAGEN consortium.

Supplementary Material

# Supplementary Methods

## Phenotype Evaluated

While other studies focusing on adolescent alcohol abuse used a less stringent cutoff (Chung et al., 2000);(Knight et al., 2003);(Fairlie et al., 2006)), the more stringent cut-off of 8 was chosen as this was more widely validated across different studies (see (Reinert and Allen, 2007) for a review). Questionnaires were given to individuals, at 14, 16 and 18 years old. While there were missing data at various times for some participants, for the purpose of this analysis, if an individual’s genetic data and AUDIT data were given at one time point, the individual was included in this study.

## Set-Based Test

To determine the relevant SNPs for our analysis, a Set-Based test was performed using PLINK1.9. For a detailed explanation of how the analysis works, see the PLINK 1.9 manual (https://www.cog-genomics.org/plink/1.9/assoc#set). In this project, three set-based tests were carried using parameters of varying stringencies. The parameters that were adjusted between the tests were p-value for significant variants between tests, r^2^ of variant pairs, and maximum set size. Data in all three set-based test underwent 10, 000 label-swapped permutation as well, using the --perm function in PLINK1.9. The first done, was the default test in PLINK1.9, with a p-value of 0.05, r^2^ of 0.5, and a set-max of 5, the second test had a p-value of 0.05, r^2^ of 0.3, and set-max of 3, while test 3 had a p of 0.01, r^2^ of 0.1 and set-max of 2. Tests 2 and 3 were more stringent, and were run to challenge the data, to ensure robustness of our results. The three set-based tests were run, with varying results (Suppl. Table1). Sixty nine SNPs appearing across five cannabinoid-related genes were analyzed for their relation to AUDIT scores. In the first set-based test, 9 SNPS returned with nominal p values of <0.05, of which 7 also passed linkage disequilibrium (LD) criterion. Through the first set-test criterion, only the CNR1 gene-set was significantly associated to an AUDIT score of eight or more (p=0.022). Within this set, only rs9353525 that was significantly and independently related to SUD. In the second set-based test, the same 9 SNPS returned with nominal p values of <0.05, of which, 5 SNPs passed the LD criterion. Again, only CNR1 was significantly associated to an AUDIT score of greater than seven (p=0.03). Finally, 4 SNPs returned with a nominal p value <0.01, in the third test, with 2 SNPs passing LD criterion. No genes remained significant after the third set-based test. As mentioned above, the 7 SNPs that had nominal p values of <0.05, in the first set-based test, and that passed LD criterion (r2<0.5) were extracted and only these were analyzed in the case-control, model analysis and logistic regression [For summary of set-based test, see Suppl. Table 6].

## Case-Control Analysis

Our first case/control association analysis was done using Fisher’s exact test, through PLINK1.9. SNPs selected for this analysis were those that were both significant and independent in the first set-based tests. Cases were considered if an AUDIT score of eight or more was reported. Four case-control analyses were run. In the first, cases were considered if an AUDIT of eight was reached at any time point (ALL); if individuals scored greater than seven at multiple time points, the duplicate data were removed. The other analyses were done at each time point; one for age 14, one for 16 and one for 18. Analysis were adjusted for multiple tests with false discovery rate correction, using the --adjust function in the PLINK1.9 program, and false discovery rate (FDR) values are reported.

## Genetic Analysis of Population Stratification

A principal components analysis based on the variance-standardized relationship matrix and displayed the 20 first genetic dimensions and associated-eigenvalues (*supplemental figure 1*) was performed. To avoid confounding factors related to ancestry the 6 first ancestry components were used as covariables in the logistic regression.

## Covariables

A logistic regression was done on the SNPs that remained significant after correction for multiple tests. Our regressions had sex, the first six components of the MDS plot, parental alcohol abuse, and parental education as co-variables. Parental education was taken from self-report answers within the European School Survey Project on Alcohol and Other Drugs (ESPAD+) questionnaire administered at the first (fourteen years old) and second time point (sixteen years old) in IMAGEN. Alcohol abuse in parents was measured using the AUDIT information obtained at the first two time points in IMAGEN. If ESPAD+ and AUDIT information were missing at the 18 year old time point, the most complete and recent information was used at this time point. For our analyses that considered AUDIT information without a regard to time, the information used in the 14 and 16 year questionnaires were mixed, so that if a parent had signalled a DRINKING issue on AUDIT at any time, they were flagged as such. Moreover if parental information was missing, individuals were not included in the logistic regression.

## Seattle Seq Annotations of SNPs

We used SeattleSeq (http://snp.gs.washington.edu/SeattleSeqAnnotation137/) to annotated SNPs in this study (Supplementary table 2).

The following annotations used in supplementary table 2 (information extract of SeattleSeq website) :

--- Name SNPs

--- chromosome (input from the user)

--- position (hg18) (input from user, location on the chromosome, hg18, 1-based)

--- position (hg19) (input from user, location on the chromosome, hg19, 1-based)

--- Major allele (Major allele frequency refers to the frequency at in an IMAGEN)

--- Minor allele (Minor allele frequency refers to the frequency at in an IMAGEN)

--- chimp. Allele (column chimpAllele: UCSC alignments)

--- Gene region (Name gene observed for SNPs)

--- FunctionGVS (GVS class of variation function, using only hg19 and your submitted alleles; see [description](http://snp.gs.washington.edu/SeattleSeqAnnotation137/HelpInputFiles.jsp#GVS-FUNCTION))

--- Function in protein (Description of genetic variation for amino acids in protein)

--- PolyPhen Prediction (column polyPhen: amino acid substitution impacts)

--- Grantham Score (column granthamScore: the Grantham score of any amino acid changes, as per [Grantham (1974) Science](http://www.sciencemag.org/content/185/4154/862.long), Table 2)

--- Conservation Score phastCons (column scorePhastCons: UCSC, 46 placental mammalian species, range of 0 to 1, with 1 being the most conserved)

--- Conservation Score GERP (column consScoreGERP: rejected-substitution score from the program GERP, Stanford University, range of -12.3 to 6.17, with 6.17 being the most conserved)

--- CADD C Score (column scoreCADD: phred-like Combined Annotation Dependent Depletion scores from Kircher et al., University of Washington, range 0 though 99)

--- HapMap Frequencies (3 columns AfricanHapMapFreq, EuropeanHapMapFreq, AsianHapMapFreq: African, European, and Asian, in percent)

--- Has Genotypes (column hasGenotypes: whether dbSNP has genotypes available for the variation)

--- dbSNP Validation (column dbSNPValidation: dbSNP validation status codes, dealing with e.g. whether the variation has been seen at least twice)

--- Repeats (2 columns repeatMasker and tandemRepeat)

--- Clinical Association (column clinicalAssociation: links to NCBI pages and PubMed)

--- Distance to Nearest Splice Site (column distanceToSplice: how close the variation is to a splice site)

--- CpG Islands (column cpgIslands: whether in a region where CpGs are present at a high level, from the UCSC genome annotation database)

--- NHLBI ESP Allele Counts (column genomesESP: the allele counts observed in the Exome Sequencing Project, optionally split by two ancestries)

--- ExAC Allele Counts (column genomesExAC: the allele counts of the Exome Aggregation Consortium, optionally split by 7 populations)

# Supplementary Results

We performed case-control analysis of the IMAGEN cohort, where cases are considered if an individual scored eight or more on AUDIT at 14, 16, or 18 years of age. At age 14, none of the 7 SNPs were significantly associated with AUD (p>0.05) [*Table S17*]. At age 16, one SNP was significantly associated to case-control status; rs9343535 (p=0.012, OR=0.656) however it did not remain significant after FDR correction [*Table S17*]. Finally, at age 18, 4 SNPs were significantly associated to case-control status group membership: rs782446 in *MGLL* (p=0.04, OR=0.813), rs484061 in *MGLL* (p=0.014, OR=0.814), rs507961 in *MGLL* (p=0.004, OR=0.742), rs9353525 in *CNR1* (p=0.025, OR=0.748). After correction for multiple tests, none remained significant [*Table S17*].

Logistic models were done for both SNPs, at each time point, as well as for any positive screen for AUD (ALL) analysis. After controlling for the effects of the first six principal components, sex, parental AUDIT scores (at any time) and parental education, both rs9353525 and rs507961 were still significantly associated with positive AUDIT screen in the ALL analysis [Table 3] (p<0.01), with both SNPs minor allele acting as protective factors (OR<1). For complete results of logistic regression see *Table S9.*

# Supplementary Figures

**Supplementary Figure 1. Principal Component Analysis of Ancestry IMAGEN**

Supplementary figure 1 Illustrations to ancestries information in Imagen. A) Distribution of Eigenvalues by principal components calculated on genetic distances in Imagen. B) Multidimensional scaling plots of the two first principal components.

# Supplementary Tables

See Attached XLS file

# Supplementary Bibliography

1. Chung T, Colby SM, Barnett NP, Rohsenow DJ, Spirito A, Monti PM. Screening adolescents for problem drinking: performance of brief screens against DSM-IV alcohol diagnoses. J Stud Alcohol. 2000 Jul;61(4):579–87.

2. Knight JR, Sherritt L, Harris SK, Gates EC, Chang G. Validity of brief alcohol screening tests among adolescents: a comparison of the AUDIT, POSIT, CAGE, and CRAFFT. Alcohol Clin Exp Res. 2003 Jan;27(1):67–73.

3. Fairlie AM, Sindelar HA, Eaton CA, Spirito A. Utility of the AUDIT for screening adolescents for problematic alcohol use in the emergency department. Int J Adolesc Med Health. 2006 Mar;18(1):115–22.

4. Aalto M, Alho H, Halme JT, Seppä K. AUDIT and its abbreviated versions in detecting heavy and binge drinking in a general population survey. Drug Alcohol Depend. 2009 Jul 1;103(1–2):25–9.

5. Reinert DF, Allen JP. The alcohol use disorders identification test: an update of research findings. Alcohol Clin Exp Res. 2007 Feb;31(2):185–99.
